# Perceived Impact of a Junior–Senior Inpatient Team Model on Clinical Workflow, Supervision, and Workload in a Tertiary Gastroenterology Department: A Mixed-Methods Study

**DOI:** 10.3390/jcm15041632

**Published:** 2026-02-21

**Authors:** Akira Uchiyama, Hiroo Fukada, Tsutomu Takeda, Hirofumi Fukushima, Maki Tobari, Dai Ishikawa, Toshio Fujisawa, Kenichi Ikejima, Akihito Nagahara, Hiroyuki Isayama

**Affiliations:** Department of Gastroenterology, Juntendo University School of Medicine, 2-1-1 Hongo, Bunkyo-ku, Tokyo 113-8421, Japan

**Keywords:** team-based inpatient care, workload distribution, clinical supervision

## Abstract

**Background:** In many inpatient settings, physician coverage is organized around single-attending responsibility, which can create challenges in supervision and workload distribution, particularly in procedurally intensive environments. To address these issues, our department introduced a junior–senior inpatient team model in which multiple physicians jointly share responsibility for hospitalized patients. This study examined physicians’ perceptions of how this restructuring influenced clinical workflow, supervision, and workload. **Methods:** We performed a mixed-methods cross-sectional survey two months after implementation. Twenty-two physicians (13 junior, 9 senior) completed five-point Likert-scale items and open-ended questions. Responses were analyzed using non-parametric group comparisons. Qualitative comments were examined thematically to identify recurring perspectives on supervision and workload. **Results:** Junior physicians reported more favorable perceptions across several domains. Significant differences between junior and senior physicians were observed for reassurance during off-site duties (*p* = 0.013) and perceived reduction in burden when managing critically ill patients (*p* = 0.002). Qualitative findings indicated that junior physicians experienced greater shared responsibility and easier access to consultation, whereas senior physicians described increased supervisory demands, responsibility extending beyond subspecialty areas, and heavier weekend or holiday duties. Both groups emphasized the importance of flexible patient redistribution during staffing variability. **Conclusions:** The junior–senior inpatient team model was associated with improved perceived accessibility of supervision and collective support for junior physicians while increasing supervisory demands on senior staff. These findings suggest the potential importance of workload-sensitive implementation strategies and explicit role definition when introducing physician team–based coverage in high-acuity inpatient settings.

## 1. Introduction

A stable patient–physician relationship is central to outpatient medicine, where continuity is typically maintained through the longitudinal responsibility of an individual attending physician [1,2]. In contrast, inpatient care requires continuous monitoring, rapid decision-making, and coordination across multiple professionals. In high-acuity environments—characterized by frequent clinical deterioration, urgent procedures, and unpredictable admissions—models that rely on single-physician responsibility may strain supervision capacity, situational awareness, and workload sustainability [3,4,5].

Team-based approaches have therefore been promoted as mechanisms to enhance reliability of oversight and distribute cognitive and operational demands. [3,4,5,6]. Within multidisciplinary hospital practice, such models typically involve collaboration among physicians, nurses, pharmacists, and allied professionals. However, even when multidisciplinary structures are well established, the internal organization of physician responsibility often remains centered on an individual attending. As a result, escalation pathways, availability for consultation, and continuity during physician absence may remain vulnerable [2,7].

These tensions are particularly relevant in gastroenterology. In many Japanese tertiary centers, gastroenterologists simultaneously maintain responsibility for inpatient management, outpatient clinics, endoscopic procedures, interventional support, and emergency coverage. This breadth of practice may intensify the impact of physician absence and increase reliance on informal supervisory mechanisms [8,9]. Under such conditions, redesigning coverage toward shared physician responsibility has been considered a potential strategy to strengthen internal support while preserving existing multidisciplinary collaboration [3,6]. To assist international readers in understanding this structural background, an overview of the division of gastroenterology responsibilities in our setting, compared with commonly described overseas arrangements, is presented in Table 1.

One such approach is the junior–senior inpatient team model, in which physicians of different experience levels jointly manage hospitalized patients within a defined team structure. Although conceptually attractive, evidence describing how physicians experience this restructuring in real clinical environments remains limited. In particular, it is unclear how junior and senior physicians perceive changes in supervision, responsibility distribution, and day-to-day workflow following implementation. 

Accordingly, the present study employed a mixed-methods design to evaluate physicians’ perceptions of the benefits and challenges associated with introducing a junior–senior inpatient team model in a tertiary gastroenterology department. By integrating quantitative ratings with qualitative insights, we sought to better understand how organizational restructuring may influence perceived supervision, workload, and clinical support.

## 2. Methods

### 2.1. Study Design and Setting

This mixed-methods cross-sectional survey study was conducted in the Department of Gastroenterology at Juntendo University Hospital, a high-volume tertiary academic medical center with approximately 1050 beds, including a gastroenterology ward with a nominal capacity of 95 beds, which frequently operates beyond its intended capacity. The department manages a broad spectrum of conditions such as gastrointestinal bleeding, hepatobiliary emergencies, oncologic care, and advanced endoscopic interventions. In addition to inpatient care, physicians routinely maintain outpatient and procedural responsibilities, resulting in frequent competing clinical demands.

Prior to October 2021, inpatient management followed a single-attending physician model supported by subspecialty conferences and weekly professor-led rounds. On 1 October 2021, the department transitioned to a physician-based team structure, the junior–senior inpatient team model (hereafter the junior–senior model). This restructuring aimed to improve workload sustainability, strengthen supervision, and ensure reliable coverage during physician absence, supported by departmental leadership and aligned with national work-style reform policies.

Under the junior–senior model, care was delivered by teams of three to four physicians, typically a senior physician, a junior physician, and a rotating resident. Members were sometimes absent because of other duties; the goal was continuous team availability rather than equal individual loads. Junior physicians conducted day-to-day management, while senior physicians provided oversight, cross-coverage, and responsibility for complex decisions. Escalation occurred through direct consultation within the team. Multidisciplinary collaboration with nurses, pharmacists, and allied professionals remained unchanged.

The survey was administered about two months after implementation (8–10 November 2021) to capture early perceptions of supervision, workload distribution, and operational impact. Because assignments varied across schedules, comparable per-respondent inpatient week data were not consistently recorded; therefore, standardized exposure measures could not be calculated.

### 2.2. Participants

All 28 physicians assigned to inpatient ward responsibilities during the survey period were invited to participate. Twenty-two physicians completed the questionnaire (response rate 78.6%). Participation was voluntary, and no incentives were offered. Physicians in postgraduate years (PGY) 1–2 were not included because they rotated under direct supervision and did not hold independent responsibility for inpatient management within the department.

To protect anonymity, detailed demographic information such as graduation year or total clinical experience was not collected.

### 2.3. Informed Consent Statement

This project was conducted as an internal quality-improvement initiative. Under institutional policy, anonymous surveys of healthcare providers that do not collect identifiable personal information may be exempt from formal ethical review. Participation was voluntary, and responses were anonymous to investigators and departmental leadership. Completion and submission of the questionnaire were considered to indicate implied informed consent.

### 2.4. Classification of Physician Experience Level

Participants self-identified their training level. For analysis, physicians were categorized as junior (PGY 3–5) or senior (PGY ≥ 6). These definitions reflected functional responsibilities within the team structure and were applied consistently across analyses.

### 2.5. Survey Instrument

The questionnaire was developed for this institutional initiative. Item generation was based on operational challenges repeatedly discussed during pre-implementation meetings among faculty members and trainees. Domains included supervision, workload, patient safety perceptions, procedural opportunities, and coverage during off-site duties. Prior to distribution, the draft survey was reviewed by representative junior and senior physicians for clarity, relevance, and interpretability, and minor wording adjustments were made accordingly. The final instrument is provided in Supplementary Questionnaire in Appendix A. The survey was administered in Japanese, the routine working language of the department. Because all participants were native speakers, translation validation procedures were not required.

### 2.6. Quantitative Measures

Closed-ended items used a five-point Likert scale ranging from strongly disagree to strongly agree. For analysis, responses were converted to a symmetrical numerical scale from −2 to +2. Although Likert data are ordinal, such transformation is widely used in perception research to preserve directional interpretation while enabling group comparison. Given the exploratory nature of the study and modest sample size, non-parametric approaches were prioritized when distributional assumptions for parametric testing were uncertain.

### 2.7. Qualitative Measures

Free-text fields captured additional perspectives regarding benefits, challenges, and suggested improvements. Qualitative analysis followed an inductive thematic approach. Two investigators independently reviewed responses, generated preliminary codes, and met to reconcile discrepancies through discussion until consensus was achieved. Because the objective was interpretive understanding rather than formal theory construction, statistical measures of inter-coder agreement were not calculated. Coding and organization of themes were performed using standard spreadsheet software.

### 2.8. Mixed-Methods Integration

Integration of quantitative and qualitative components occurred during interpretation. Thematic findings were used to contextualize numerical trends and to clarify how physicians understood observed differences between junior and senior groups.

### 2.9. Statistical Analysis

Quantitative variables are presented as medians with interquartile ranges or as counts and percentages, as appropriate. Comparisons between junior and senior physicians were performed using the Mann–Whitney U test depending on distributional characteristics. All tests were two-sided, and *p* < 0.05 was considered statistically significant. Analyses were conducted using SigmaPlot version 15 (Systat Software, San Jose, CA, USA). Given the exploratory and hypothesis-generating nature of the study and the modest sample size, formal adjustments for multiple comparisons (e.g., Bonferroni correction) were not applied. Therefore, *p*-values should be interpreted with appropriate caution.

## 3. Results

### 3.1. Respondent Characteristics

Participant characteristics are summarized in Table 2. To preserve respondent anonymity, detailed demographic information—such as graduation year and total years of clinical experience—was not collected. During the survey period, inpatient care was delivered across eight teams, each typically responsible for approximately 13 patients, yielding an overall census of around 110 hospitalized patients. Each team also managed approximately one to two ICU-level patients at any given time. Scheduled admissions averaged eight per day, with an additional two emergency admissions per day.

### 3.2. Perceived Changes After Implementation of Junior–Senior Inpatient Teams

#### 3.2.1. Team Functioning (Figure 1)

**Figure 1:**
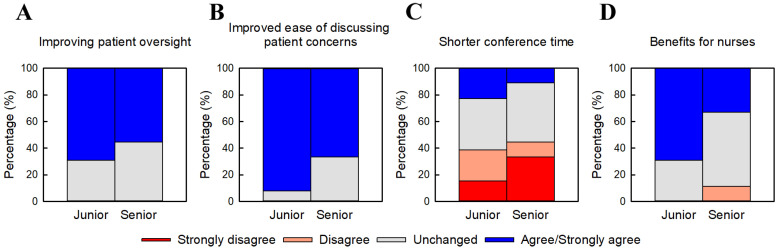
Perceived team functioning under the junior–senior inpatient team model. Physicians’ evaluations of four aspects of team functioning after the transition from individual physician responsibility for inpatient care to junior–senior inpatient teams. (**A**) Improved patient oversight. (**B**) Improved ease of discussing patient concerns. (**C**) Shorter conference time. (**D**) Perceived benefits for nurses. Responses are presented as percentage distributions on a five-point Likert scale. Percentages are based on junior physicians (n = 13) and senior physicians (n = 9). Numerical scores (–2 to +2) were compared between groups using the Mann–Whitney Rank Sum Test. Although junior physicians generally reported more favorable perceptions across items, none of the differences reached statistical significance.

In this study, team functioning was operationally represented by survey items addressing patient oversight, accessibility of discussion, conference burden, and perceived benefits for nurses. Figure 1 summarizes physicians’ evaluations of four aspects of team functioning following the transition to junior–senior inpatient teams. Overall, both junior and senior physicians reported generally favorable impressions, although the degree of endorsement varied across items. For patient oversight (Figure 1A), positive perceptions were reported by 69.2% (9/13) of junior physicians and 55.6% (5/9) of senior physicians. Junior physicians more frequently reported ease in discussing patient management issues within the team (Figure 1B). The full distribution of responses across all Likert categories is provided in Supplementary Table S1. Perceptions regarding reduced conference time (Figure 1C) were limited in both groups. These evaluations reflect physicians’ perceptions of how the team structure influenced interprofessional workflow rather than direct reports from nursing staff. Regarding perceived benefits of the team system for nurses (Figure 1D), junior physicians reported more favorable evaluations than senior physicians. Across all four domains, junior physicians consistently reported higher ratings than senior physicians.

#### 3.2.2. Clinical Benefits of Junior–Senior Inpatient Teams (Figure 2)

**Figure 2:**
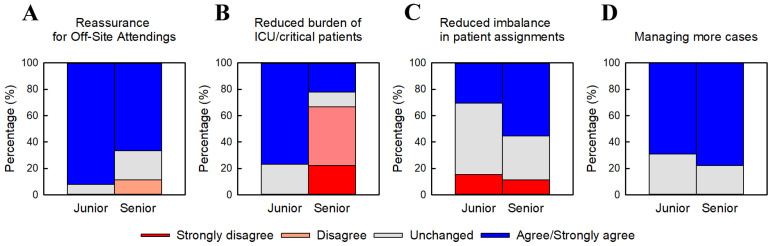
Clinical benefits of the junior–senior inpatient team model. Physicians’ perceptions of four clinical benefits associated with junior–senior inpatient team model. (**A**) Reassurance when physicians are off-site. (**B**) Reduced burden when managing ICU or severely ill patients. (**C**) More balanced patient assignment. (**D**) Increased exposure to a broader range of cases. Responses are presented as percentage distributions on a five-point Likert scale. Numerical scores (–2 to +2) were compared between junior physicians (PGY 3–5) and senior physicians (PGY ≥ 6) using the Mann–Whitney Rank Sum Test, as appropriate. Junior physicians reported significantly higher scores for items (**A**) (*p* = 0.013) and (**B**) (*p* < 0.01), whereas items (**C**) and (**D**) showed no statistically significant differences between groups. Percentages are based on junior physicians (n = 13) and senior physicians (n = 9).

Figure 2 summarizes physicians’ perceptions of four clinical benefits associated with junior–senior inpatient teams. Junior physicians consistently reported higher scores than senior physicians, although the magnitude and statistical significance of differences varied across items. Reassurance was interpreted by respondents as the availability of immediate consultation, decision support, and cross-coverage within the team when the primary physician was engaged elsewhere. For reassurance when physicians were off-site (Figure 2A), junior physicians reported significantly higher scores than senior physicians (median 1.0 vs. 0; *p* = 0.013). Respondents generally interpreted reduced burden as the sharing of cognitive and emotional load rather than the elimination of clinical responsibility. Junior physicians also reported a significantly lower perceived burden when managing ICU or severely ill patients under the junior–senior inpatient team model (Figure 2B; *p* = 0.002). Qualitative responses suggested that some of this reduction for junior physicians may have corresponded to increased supervisory demand for senior staff. Perceptions regarding whether junior–senior inpatient teams reduced uneven distribution of patient assignments (Figure 2C) differed between groups but did not reach statistical significance (*p* = 0.334). For the item assessing whether the system enabled physicians to experience a greater number of inpatient cases (Figure 2D), both junior and senior physicians expressed strong agreement, with no significant difference between groups (*p* = 0.744).

#### 3.2.3. Perceived Benefits: Qualitative Themes (Table 3)

A total of 12 physicians provided free-text comments (7 junior, 5 senior). Qualitative analysis provided additional insight into perceived benefits of junior–senior inpatient teams (Table 3). Junior physicians commonly identified the following advantages:

- Broader exposure to a diverse range of inpatient cases;
- Reduced individual burden when managing critically ill or ICU-level patients;
- Improved ability to manage ward duties after external duties;
- Increased time available for self-directed learning and academic activities.

Both junior and senior physicians reported that shared discussions within the team contributed to perceptions of improved patient safety.

**Table 3:** Themes of perceived benefits associated with the junior–senior inpatient team model.

Benefits Category	Junior Physicians	Senior Physicians	Representative Comments
Increased clinical exposure	Identified	Not identified	I was able to experience a greater variety of cases than before.
Reduced burden of managing critically ill or ICU-level patients	Identified	Not identified	Managing critically ill patients became less overwhelming.
Improved ability to complete ward responsibilities after external duties	Identified	Not identified	Ward tasks were easier to finish after returning from afternoon outreach duties.
Increased time available for self-directed learning and academic activities	Identified	Not identified	I had more time available for learning and academic tasks.
Enhanced patient safety through shared clinical oversight	Identified	Identified	Team discussions improved risk management and contributed to safer patient care.

### 3.3. Workflow and Workload Changes

#### 3.3.1. Quantitative Evaluations (Figure 3)

**Figure 3:**
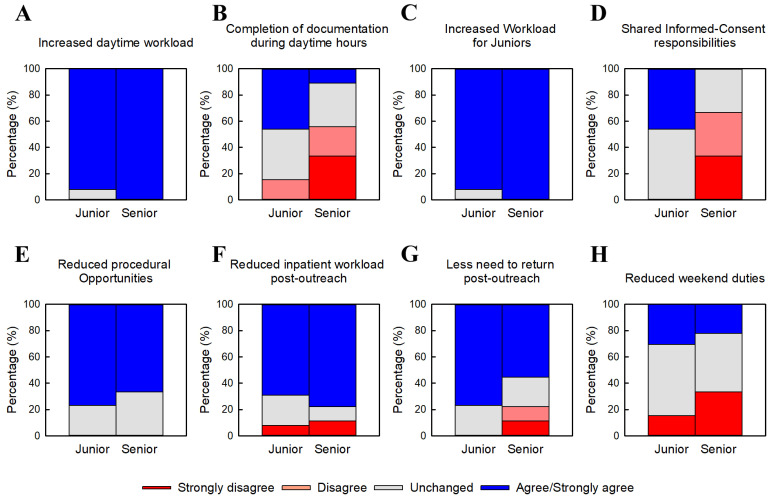
Workload-related and operational evaluations. Physicians’ evaluations of eight workload-related and operational aspects following the transition to junior–senior inpatient teams. (**A**) Increased daytime workload. (**B**) Completion of documentation during daytime hours. (**C**) Increased workload for junior physicians. (**D**) Reduced responsibilities for informed consent. (**E**) Reduced procedural opportunities. (**F**) Reduced ward workload after return from outside outreach. (**G**) Reduced need to return to the ward after afternoon outreach. (**H**) Reduced weekend ward duties. Responses are presented as percentage distributions on a five-point Likert scale. Percentages are based on junior physicians (n = 13) and senior physicians (n = 9). Numerical scores (–2 to +2) were compared between junior physicians (PGY 3–5) and senior physicians (PGY ≥ 6) using the Mann–Whitney Rank Sum Test as appropriate. Significant group differences were observed for items (**B**) and (**D**), with junior physicians reporting more favorable evaluations (*p* = 0.01 and *p* < 0.001, respectively). No statistically significant differences were found for items (**A**,**C**,**E**,**F**,**G**), or (**H**).

Figure 3 presents physicians’ evaluations of eight workload-related and operational aspects following the transition to junior–senior inpatient teams. For increased daytime workload (Figure 3A), both junior and senior physicians predominantly selected agree or strongly agree, with no significant difference between groups (*p* = 0.085). Junior physicians reported significantly more favorable perceptions regarding completion of documentation during daytime hours (Figure 3B), reflected by higher median scores (*p* = 0.011). Perceptions of increased daytime workload specifically for junior physicians (Figure 3C) were positive in both groups, with no significant difference (*p* = 0.365). Significant group differences were observed for reduced responsibilities related to informed consent (Figure 3D), with junior physicians reporting markedly more favorable evaluations (*p* < 0.001).

In contrast, perceptions regarding reduced procedural opportunities due to ward busyness (Figure 3E) and reduced ward duties after returning from off-site outreach (Figure 3F) were similarly positive in both groups, with no significant differences (*p* = 0.942 and *p* = 1.000, respectively). For reduced need to return to the ward after afternoon outreach (Figure 3G), junior physicians tended to report more favorable perceptions, although this difference did not reach statistical significance (*p* = 0.106). Evaluations of reduced weekend ward duties (Figure 3H) showed mixed responses among both junior and senior physicians, with no significant differences between groups.

#### 3.3.2. Remaining Operational Challenges (Figure 4)

**Figure 4:**
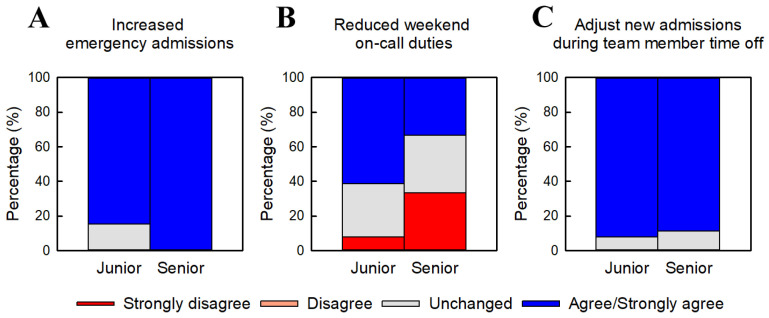
Remaining challenges associated with the junior–senior inpatient team model. Physicians’ perceptions of remaining challenges following the transition to junior–senior inpatient teams. (**A**) Increased emergency admissions. (**B**) Reduced weekend on-call duties. (**C**) Need for adjusting inpatient assignments during staff absences. Responses are presented as percentage distributions on a five-point Likert scale. Percentages are based on junior physicians (n = 13) and senior physicians (n = 9). Numerical scores (–2 to +2) were compared between junior physicians (PGY 3–5) and senior physicians (PGY ≥ 6) using the Mann–Whitney Rank Sum Test. Senior physicians reported significantly higher scores for item (**A**) (*p* = 0.02), whereas items (**B**) and (**C**) showed no statistically significant differences between groups.

Figure 4 summarizes physicians’ perceptions of remaining challenges following implementation of junior–senior inpatient teams. For increased emergency admissions (Figure 4A), both junior and senior physicians reported higher workload, with senior physicians indicating a significantly greater perceived impact than junior physicians. Objective pre–post admission counts were not collected; therefore, this item reflects perceived change rather than measured variation in admission volume. Perceptions regarding reduced weekend on-call duties (Figure 4B) differed between groups, with junior physicians tending to report more favorable evaluations; however, this difference did not reach statistical significance. For the need to adjust inpatient assignments during staff absences (Figure 4C), both junior and senior physicians expressed strong agreement, with no significant difference between groups. In practice, when physicians were absent due to conferences or illness, new admissions were preferentially directed away from those teams, and patient allocation was adjusted to prevent excessive concentration of workload.

#### 3.3.3. Perceived Challenges: Qualitative Themes (Table 4)

Qualitative analysis further clarified key challenges associated with junior–senior inpatient teams (Table 4). Both junior and senior physicians described daytime workload as “very busy,” reflecting a shared perception of increased clinical demands. Junior physicians reported fewer opportunities to participate in procedures and noted that increased emergency admissions substantially added to their workload. In contrast, senior physicians highlighted difficulties in supervising patients outside their own subspecialty areas and expressed concerns regarding increased weekend and holiday responsibilities. Across both groups, physicians emphasized the need for adjustment of patient assignments and workload distribution during periods of staff absence.

**Table 4:** Themes of perceived challenges associated with the junior–senior inpatient team model.

Challenges Category	Junior Physicians	Senior Physicians	Representative Comments
Increased daytime workload	Identified	Identified	The daytime workload has increased and feels very busy.
Increase in emergency admissions	Identified	Not identified	Emergency admissions have increased and added to the workload.
Reduced opportunities to participate in procedures	Reported	Not identified	I have fewer chances to take part in procedures, such as endoscopy.
Heavy weekend/holiday workload	Not identified	Identified	Weekend and holiday duties have increased for senior physicians.
Difficulty supervising patients outside one’s specialty	Not identified	Identified	It is challenging to supervise patients outside my specialty.
Need for admission and workload adjustments during member absences	Identified	Identified	Teams need additional support when members are on leave.

### 3.4. Workload Expectations and Structural Preferences (Table 5) 

As shown in Table 5, physicians reported a preferred median patient load of 10 patients per team (IQR, 10–12), while 15 patients (IQR, 15–15) was most commonly identified as the upper acceptable limit. Regarding structural preferences, only a minority of physicians favored regular reorganization of teams (defined as rotation-based reassignment of membership approximately every 2–3 months) (5 respondents), whereas most preferred to maintain the existing team structure (17 respondents). Similarly, fewer physicians supported consolidating teams into larger units (8 respondents), while the majority favored retaining the current configuration (14 respondents).

**Table 5:** Workload expectations and structural preferences under the junior–senior inpatient team model.

Survey Item	Median (IQR)
Preferred number of patients per team	10.0 (IQR 10–12)
Upper limit of manageable patients per team	15.0 (IQR 15–15)
Preferred regular team reorganization	Yes (n = 5), No (n = 17)
Preference for fewer teams with redistributed physicians	Yes (n = 8), No (n = 14)

IQR; interquartile range.

## 4. Discussion

This study evaluates perceived, rather than objectively measured, changes in supervision, workload, and patient safety following the transition from an individual-attending structure to a junior–senior inpatient team model. The findings therefore represent how physicians interpreted the organizational redesign and should not be considered direct evidence of improvement in clinical outcomes or safety performance.

Several clear quantitative patterns emerged. Junior physicians reported significantly greater reassurance during off-site duties (*p* = 0.013) and a stronger perception of reduced burden when caring for critically ill patients (*p* = 0.002). In contrast, qualitative narratives from senior physicians consistently emphasized increased supervisory responsibility [10,11], extension of oversight beyond subspecialty expertise, and heavier weekend or holiday obligations. Taken together, these convergent data suggest that the model redistributed responsibility across experience levels rather than reducing the total workload within the system [5,9,12], a phenomenon described in prior work on team-based coverage and shared accountability [5,12]. Qualitative challenges matched the quantitative patterns—greater daytime workload, emergency demand, and concentrated supervisory responsibility—supporting internal consistency. They also showed that benefits for junior physicians came with redistributed supervisory and cognitive demands for senior physicians.

Interpreting these results requires consideration of the structural context of Japanese gastroenterology practice. Gastroenterologists in tertiary centers frequently maintain simultaneous commitments to inpatient care, outpatient clinics, endoscopic procedures, emergency coverage, and peri-procedural management. The junior–senior framework therefore operated as a ward-centered physician coverage system embedded within ongoing procedural demands, rather than separating inpatient and specialty services as seen in many hospitalist-based models [3,6,7]. Such complexity has been associated with increased vulnerability to workload strain and burnout [8,9].

From a governance and healthcare leadership perspective, restructuring physician coverage can be viewed as a practical response to pressures on sustainability and the need for reliable care in high-acuity systems [4,13]. This interpretation is consistent with emerging evidence-based management frameworks, which emphasize leadership commitment, workforce durability, and data-informed organizational redesign in complex healthcare environments [14]. Within this broader context, the Junior–Senior Inpatient Team may be understood not simply as an operational modification, but rather as a strategic response aimed at maintaining service stability under real-world workforce constraints. Previous literature suggests that team-based structures are often introduced to enhance continuity, distribute cognitive demand, and reinforce safety through collective oversight [3,5].

Internationally, hospitalist and multiple-attending models often separate inpatient care from outpatient and procedural duties, enabling continuous supervision and clearer escalation pathways [3,6]. In contrast, Japanese gastroenterologists typically maintain simultaneous responsibilities for wards, clinics, and endoscopy. Within this context, the Junior–Senior framework functioned as a ward-centered physician coverage system embedded within ongoing procedural demands rather than a structural separation of services. Thus, in our setting, team implementation added shared oversight without reducing baseline workload. This difference may explain why junior physicians experienced greater reassurance, whereas senior physicians perceived increased supervisory demands [5,9].

Importantly, multidisciplinary collaboration was already embedded in routine care prior to implementation. Nurse–physician coordination [15], pharmacist participation [16], and structured senior-led ward rounds formed part of standard workflow. The reform therefore added a new layer of shared physician responsibility to an established multidisciplinary environment. This may explain why benefits were primarily described as improved internal consultation and reassurance, consistent with literature indicating that structured collaboration enhances perceived reliability even when formal outcomes are not directly measured [3,12].

Differences between junior and senior perceptions may also reflect sociocultural and educational traditions [17]. In hierarchical training systems, senior physicians often retain implicit ultimate accountability, whereas junior physicians may experience greater psychological safety when access to supervision becomes more explicit [18]. Concepts of graded responsibility and shared decision-making in complex environments support this interpretation [10,11]. While the model provided important advantages, the data also highlight meaningful unintended consequences. Senior physicians reported concentration of supervisory load, cross-subspecialty coverage, and intensified non-weekday duties. Junior physicians, despite improved reassurance, reported reduced access to procedural participation because ward management demands competed with training opportunities. A similar balance between support and workload redistribution has been described in prior analyses of team redesign [5,9].

Another key finding concerns patient volume. Respondents consistently indicated that approximately 10 patients per team represented a desirable census, whereas levels approaching 15 challenged situational awareness. Evidence from teamwork and safety research suggests that increasing workload can erode communication reliability and oversight capacity even in structured teams [4,5]. Thus, expansion of patient numbers without parallel resource adaptation may limit the benefit of organizational reform.

For institutions considering similar transitions, implementation should extend beyond structural change. Flexible redistribution of admissions during absences, explicit allocation of supervisory roles, and protection of procedural learning time may help prevent concentration of burden. Future evaluations would benefit from incorporating objective metrics—such as escalation patterns or safety events—in addition to perception data, an approach recommended in patient safety research [4,5].

Several limitations warrant consideration. First, the study did not include objective clinical outcomes, incident data, or direct measures of supervisory effectiveness; therefore, conclusions remain perception-based. Second, responses may have been influenced by social desirability within a hierarchical professional culture. Third, demographic and professional variables such as years of experience or subspecialty focus were not collected, limiting adjustment for confounding. Fourth, the survey was conducted early after implementation, and perceptions may change over time. Some perceptions observed shortly after implementation may reflect transitional adaptation or resistance to organizational change rather than stable indicators of long-term workflow performance. Finally, because Japanese gastroenterologists often maintain broader scopes of practice than physicians in hospitalist-based systems, generalizability to other settings may be limited.

## 5. Conclusions

Within this perception-based, cross-sectional evaluation, the junior–senior model was associated with improved perceived accessibility of supervision and enhanced collective oversight, particularly among junior physicians, while increasing supervisory demands on senior physicians. These findings reflect subjective experiences rather than objectively measured improvements in patient safety or clinical outcomes. Successful implementation of physician team–based coverage may therefore require explicit attention to sustainable workload distribution, prevention of excessive concentration of responsibility on senior staff, and clearly defined accountability structures to help mitigate potential risks of burnout and workforce attrition.

## Figures and Tables

**Table 1:** Overview of gastroenterology practice structures in Japan and overseas settings.

Scope of Practice	Japan (Our Department)	Overseas (Typical Model)
Inpatient care	✓ Gastroenterologists as primary attending physicians	◯ Managed mainly by hospitalists
Outpatient practice	✓ Managed by the same gastroenterologists	✓ GI/◯ NP-led clinics
Routine endoscopy (EGD, CS)	✓ Performed by gastroenterologists	✓ Performed by gastroenterologists
Advanced endoscopy (EMR/ESD, ERCP, EUS)	✓ Performed by gastroenterologists	◯ Subspecialized endoscopists
Emergency endoscopy (night/weekend)	✓ Gastroenterologists on call	◯ Dedicated on-call teams
IR-related procedures (PTBD, PTC, biopsy)	✓ Some performed by gastroenterologists	◯ Interventional radiologists
Sedation management	✓ Managed by gastroenterologists	◯ Anesthesiology teams
Cancer systemic therapy	✓ Managed by gastroenterologists	◯ Medical oncologists
Team structure	✓ Physician-centered	◯ Multidisciplinary, task-shifted
Overall workload concentration	✓ High individual concentration	◯ Distributed across teams

Symbols: ✓ = performed/managed by gastroenterologists; ◯ = mainly managed by other specialists or through task shifting. This table is intended to provide contextual background rather than a direct comparison between healthcare systems.

**Table 2:** Characteristics of physicians participating in the inpatient ward survey.

Variable	N (%)
Total physicians invited	28
Total respondents	22 (78.6%)
Junior physicians	13
Senior physicians	9
Years of clinical experience	Not collected *
Graduation year	Not collected *

* To maintain respondent anonymity, individual data such as exact years of experience and graduation year were not collected.

## Data Availability

The datasets generated and analyzed during the current study consist of anonymized survey responses and are not publicly available due to institutional policy restricting the release of internal quality-improvement data. However, the data are available from the corresponding author upon reasonable request and with permission from the institution.

## Supplementary Materials

The following supporting information can be downloaded at: https://www.mdpi.com/article/10.3390/jcm15041632/s1: Supplementary Table S1. Full distribution of responses across all Likert categories for Figure 1, Figure 2, Figure 3 and Figure 4, including raw counts and corresponding group sizes.

## Abbreviation

The following abbreviation is used in this manuscript:

ICU	Intensive care unit

## Appendix A. Survey Questionnaire After the Transition to the Junior–Senior Inpatient Teams

Participants were asked to rate each statement using a 5-point Likert scale ranging from −2 (strongly disagree) to +2 (strongly agree).

1. The physician-based inpatient care model improved patient oversight within the team.
2. I was able to discuss patient care within the team instead of managing patients on my own.
3. The time spent on patient conferences has decreased.
4. The physician-based inpatient care model is beneficial for ward nurses.
5. The physician-based inpatient care model makes me feel more secure when I am off-site.
6. The workload when caring for ICU or critically ill patients has decreased.
7. The imbalance in assigned patients decreased.
8. I was able to gain experience with a greater number and variety of cases.
9. The workload during daytime hours increased.
10. Under the physician-based inpatient care model, I am able to complete charting during daytime hours.
11. Junior physicians felt busier.
12. I felt that the burden of obtaining informed consent decreased.
13. Under the physician-based inpatient care model, participation in endoscopic and other procedures decreased.
14. Ward duties after afternoon off-site work decreased.
15. It became possible to go directly home after off-site duties, allowing time for self-directed learning.
16. The frequency of weekend duties decreased.
17. The number of emergency admissions increased.
18. Weekend on-call duties decreased.
19. I would like inpatient admissions to be adjusted when team physicians are resting or on leave.
20. The preferred number of patients per team? Preferred number: ___ patients.
21. The maximum number of patients per team considered manageable? Maximum number: __patients.
22. Additional comments (free-text).
