# Supplementary material for: Perceived Impact of a Junior–Senior Inpatient Team Model on Clinical Workflow, Supervision, and Workload in a Tertiary Gastroenterology Department: A Mixed-Methods Study
Source: J Clin Med. 2026 Feb 21;15(4):1632. doi: 10.3390/jcm15041632 (PMC12941908; doi:10.3390/jcm15041632)
Supplement: Supplementary file 1 [file jcm-15-01632-s001.zip › jcm-4137489-supplementary.pdf]

Supplementary Table S1

Item	Group	Strongly disagree	Disagree	Unchanged	Agree/Strongly agree
**Improved patient oversight**	Junior (n=13)	0	0	4	9
	Senior (n=9)	0	0	4	5
**Improved ease of discussing patient concerns**	Junior (n=13)	0	0	1	13
	Senior (n=9)	0	0	3	6
**Shorter conference time**	Junior (n=13)	2	3	5	3
	Senior (n=9)	3	1	4	1
**Perceived benefits for nurses**	Junior (n=13)	0	0	4	9
	Senior (n=9)	0	1	5	3
**Reassurance for off-site attendings**	Junior (n=13)	0	0	1	12
	Senior (n=9)	0	1	2	6
**Reduced burden of ICU/critical patients**	Junior (n=13)	0	0	3	10
	Senior (n=9)	2	4	1	2
**Reduced imbalance in patient assignment**	Junior (n=13)	2	0	7	4
	Senior (n=9)	1	0	3	5
**Managing more cases**	Junior (n=13)	0	0	4	9
	Senior (n=9)	0	0	2	7
**Increased daytime workload**	Junior (n=13)	0	0	1	12
	Senior (n=9)	0	0	0	9
**Completion of documentation during daytime hours**	Junior (n=13)	0	2	5	6
	Senior (n=9)	3	2	3	1
**Increased workload for junior**	Junior (n=13)	0	0	1	12
	Senior (n=9)	0	0	0	9
**Shared informed-consent responsibilities**	Junior (n=13)	0	0	7	6
	Senior (n=9)	3	3	3	0
**Reduced procedural opportunities**	Junior (n=13)	0	0	3	10
	Senior (n=9)	0	0	3	6
**Reduced inpatient workload post-outreach**	Junior (n=13)	1	0	3	9
	Senior (n=9)	1	0	1	7
**Less need to return post-outreach**	Junior (n=13)	0	0	3	10
	Senior (n=9)	1	1	2	5
**Reduced weekend ward duties**	Junior (n=13)	2	0	7	4
	Senior (n=9)	3	0	4	2
**Increased emergency admissions**	Junior (n=13)	0	0	2	11
	Senior (n=9)	0	0	0	9
**Reduced weekend on-call duties**	Junior (n=13)	1	0	4	8
	Senior (n=9)	3	0	3	3
**Adjust new admissions during team member time off**	Junior (n=13)	0	0	1	12
	Senior (n=9)	0	0	1	8
